# Evaluation of pancreatic cancer specimens for comprehensive genomic profiling

**DOI:** 10.1111/pin.13416

**Published:** 2024-03-13

**Authors:** Kota Washimi, Yukihiko Hiroshima, Shinya Sato, Makoto Ueno, Satoshi Kobayashi, Naoto Yamamoto, Chie Hasegawa, Emi Yoshioka, Kyoko Ono, Yoichiro Okubo, Tomoyuki Yokose, Yohei Miyagi

**Affiliations:** ^1^ Department of Pathology Kanagawa Cancer Center Yokohama Kanagawa Japan; ^2^ Division of Advanced Cancer Therapeutics Kanagawa Cancer Center Research Institute Yokohama Kanagawa Japan; ^3^ Center for Cancer Genome Medicine, Kanagawa Cancer Center Yokohama Kanagawa Japan; ^4^ Division of Molecular Pathology and Genetics Kanagawa Cancer Center Research Institute Yokohama Kanagawa Japan; ^5^ Department of Gastoroenterology Kanagawa Cancer Center Yokohama Kanagawa Japan; ^6^ Department of Gastrointestinal Surgery Kanagawa Cancer Center Yokohama Kanagawa Japan

**Keywords:** biopsy, neoplasms, next‐generation sequencing, pancreatic carcinoma, therapeutics

## Abstract

Inadequate specimen quality or quantity hinders comprehensive genomic profiling in identifying actionable mutations and guiding treatment strategies. We investigated the optimal conditions for pancreatic cancer specimen selection for comprehensive genomic profiling. We retrospectively analyzed 213 pancreatic cancer cases ordered for comprehensive genomic profiling and compared results from pancreatic biopsy, liver biopsy of pancreatic cancer metastases, pancreatectomy, liquid, and nonliver metastatic organ specimens. We examined preanalytical conditions, including cellularity (tumor cell count/size). The successfully tested cases were those that underwent comprehensive genomic profiling tests without any issues. The successfully tested case ratio was 72.8%. Pancreatic biopsy had the highest successfully tested case ratio (87%), with a high tumor cell percentage, despite the small number of cells (median, 3425). Pancreatic biopsy, liver biopsy of pancreatic cancer metastases, and non‐liver metastatic organ had higher successfully tested case ratios than that for pancreatectomy. Liver biopsy of pancreatic cancer metastases and pancreatectomy cases with tumor size (mm^2^) × tumor ratio (%) > 150 and >3000, respectively, had high successfully tested case ratios. The success of comprehensive genomic profiling is significantly influenced by the tumor cell ratio, and pancreatic biopsy is a potentially suitable specimen for comprehensive genomic profiling.

**Abbreviations**
Aging: time from specimen block preparation to submission for testing (day)
CDx: companion diagnostics
CGP: comprehensive genomic profiling tests
CHIP: clonal hematopoiesis with indeterminate potential
DNA: deoxyribonucleic acid
Liquid: liquid biopsy specimens
L‐biopsy: liver biopsy specimens of pancreatic cancer liver metastases
NE: not evaluable
Others: specimens collected from metastatic areas other than liver metastases
PD: Progression
PR: partial response
P‐biopsy: pancreatic biopsy specimens
P‐ope: pancreatectomy specimens
SD: stable disease
VUS: variant of unknown significance

## INTRODUCTION

The field of tumor treatment has recently witnessed a substantial advancement in the form of establishment of comprehensive genomic profiling (CGP). CGP utilizes next‐generation sequencing (NGS) techniques to analyze numerous genes within a single assay, including crucial cancer biomarkers outlined in guidelines and clinical trials, thereby aiding in selecting an optimal treatment strategy.^1^ The identification of actionable mutations using CGP has been instrumental in determining effective treatment options and potential participation in clinical trials for patients with cancer. CGP results can occasionally impact the pathological diagnosis.^2^ However, challenges, such as insufficient sample quality or quantity resulting in reports with limited information, may impede the testing.^3^, ^4^ Consequently, careful selection of specimens that do not compromise the benefits to patients with cancer is essential. Reports with limited information may not be helpful in evaluating microsatellite instability (MSI) or tumor mutational burden (TMB), potentially leading to failure in noticing actionable mutations.

**Figure 1:**
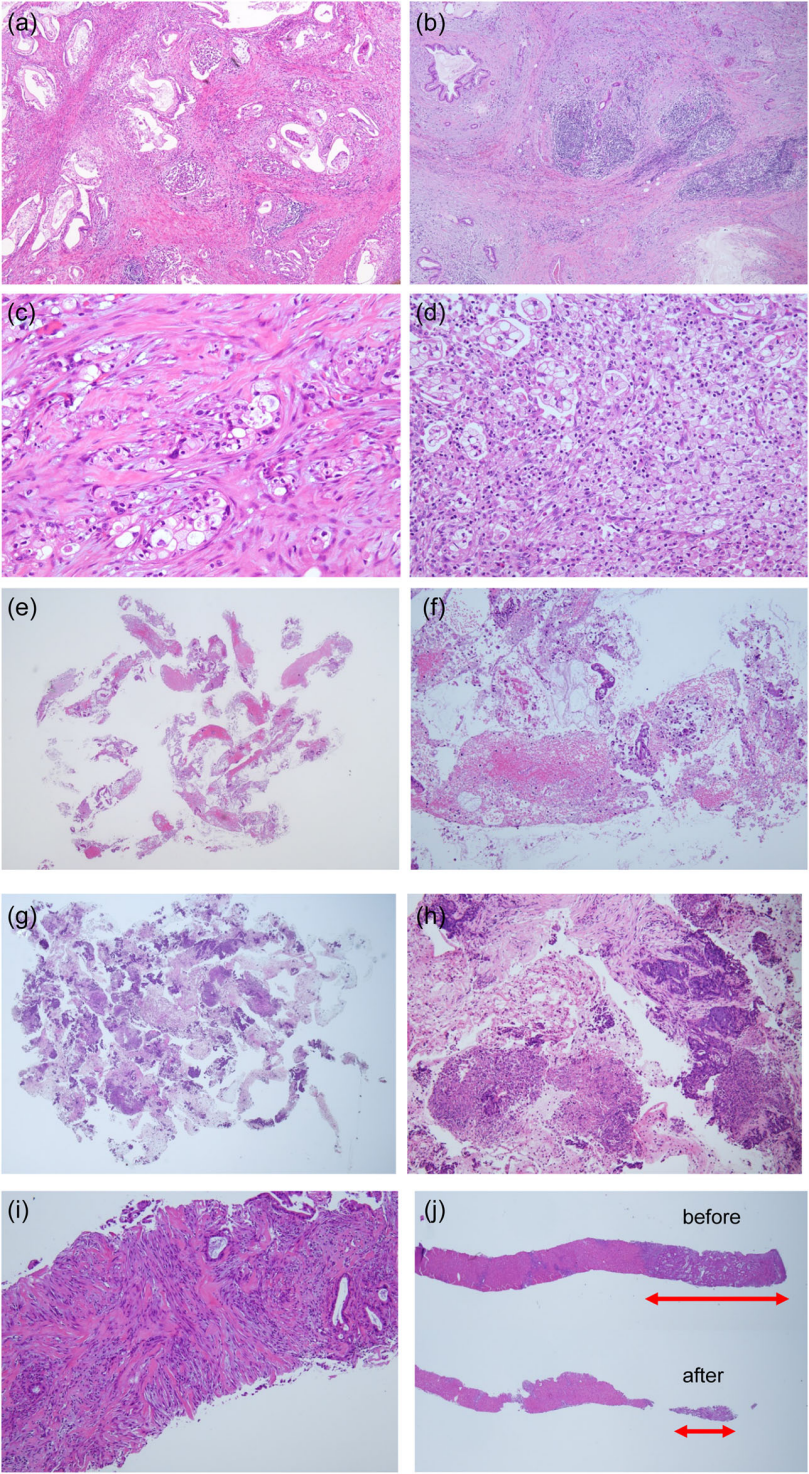
hematoxylin and eosin (HE) findings. P‐ope: (a) Adenocarcinoma with abundant stromal cell proliferation. (b) Fibrosis and lymphocytic infiltration are prominent in the pancreas. (c) Postchemotherapy. Tumor cell degeneration and fibroblast proliferation. (d) Post‐chemotherapy. Tumor cell degeneration and foci of histiocytes are observed. P‐biopsy: (e) Fragmented hemorrhage and fibrin were collected, in which a small number of cells can be observed. (f) Same case as (e). Scattered adenocarcinoma cells on a fibrin background. P‐biopsy: (g) Limited report. Tumor cells: 11417, specimen area: 30 mm^2^, tumor cell ratio: 40%, purity assessment: 49.5. (h) Same case as (a). High tumor cell counts with prominent necrosis and inflammatory cells. L‐biopsy: (i) Limited report. Active fibroblast proliferation among adenocarcinoma cells. Tumor area: 10.4 mm^2^, tumor cell ratio: 30%, purity: 20. (j) Examination not possible postsubmission. Initial and final tumor areas (red arrows) after thin sectioning: 1.6 and 0.2 mm^2^, respectively. Tumor cell ratio: 10%.

Among digestive cancers, pancreatic cancer presents the most unfavorable prognosis. Only 15%–20% of cases are organ‐confined and amenable to surgical removal, whereas 30%–40% are locally advanced, and 50%–60% involve distant metastasis.^5^ Despite the significance of CGP in guiding treatment modalities and identifying therapeutic options, a considerable number of pancreatic cancer cases are either unsuitable for testing or account for more reports with limited information than other tumor‐type cases.^6^ Notably, pancreatic cancer accounted for the highest proportion of CGP testing reports at our hospital. This could be partly attributed to a lower cellularity and tumor‐cell ratio than those in other carcinomas, likely owing to increased fibrosis and less dense tumor growth with a well‐differentiated glandular ductal architecture, even in invasive tumors (Figure 1a). In pancreatic cancer surgical specimens, preoperative chemotherapy was frequently administered, leading to tumor cell degeneration, an increased fibrosis area after tumor regression, and a perceived low tumor cell ratio owing to the infiltration of clustered lymphoid and histiocyte‐reactive inflammatory cells (Figure 1b–d). In pancreatic cancer biopsy, specimens typically yield sporadic collections of tumor cells amidst fibrin and hemorrhagic tissues (Figure 1e,f). Accordingly, we analyzed the results of pancreatic cancer CGP tests conducted at our hospital and explored the selection criteria for appropriate specimens with a high probability for successful pancreatic CGP testing.

## METHODS

### Study design and specimens

We comprehensively and retrospectively analyzed 213 cases of pancreatic cancer for which specimens were ordered to undergo CGP tests from August 2019 to December 2022. The CGP tests used were FoundationOne® CDx and FoundationOne® CDx Liquid (both Foundation Medicine). We compared five types of specimens: pancreatic biopsy specimens (P‐biopsy) obtained through aspiration, liver biopsy specimens of pancreatic cancer metastases (L‐biopsy), pancreatectomy specimens (P‐ope), liquid biopsy specimens (Liquid), and specimens collected from metastatic organ sites other than the liver (Others). The “Others” category comprised five specimens from the peritoneum, two from the ovary, one from the omentum, one from the small intestine, and one from the lung.

Specimens submitted for FoundationOne® CDx testing were required to have a minimum tumor area of 25 mm² on section and a tumor cell percentage of ≥20%. In cases where the tumor area was <25 mm², the number of sections was increased to achieve a tumor volume of 1 mm³.

This study was approved by the Research Ethics Review Committee of Kanagawa Cancer Center (approval no. 2022 epidemiology‐133). This investigation was conducted in accordance with the Declaration of Helsinki.

### Factors for analysis, tumor cellularity assessment, and categorization of results

For our analysis, we considered several factors prior to specimen submission, including the presence of chemotherapy prior to specimen collection, radiographic evaluation of chemotherapy efficacy (NE: not evaluable, PD: progression, SD: stable disease, and PR: partial response), and pathological evaluation of chemotherapy efficacy (Evans classification: I, IIa, Iib, and III). Factors involved in specimen preparation comprised formalin fixation time (in hours), type of formalin used (neutral 10% buffered, neutral 20% buffered, and 10% unbuffered), and time elapsed after the preparation of formalin‐fixed paraffin‐embedded tissue blocks to submission for testing (aging, in days).^7^, ^8^, ^9^, ^10^, ^11^, ^12^ Factors involved in the pathology included the tumor cell ratio (%), which was assessed by pathologist K.W., the tissue area size (mm^2^), as well as the number of tumor cells, which was quantified using the image analysis software Patholoscope® (MITANI Corporation).

The CGP test results were categorized as follows: cases determined unsuitable for CGP by the pathologist (not submitted), cases deemed not feasible after submission to the test (test failure), cases yielding a report with limited information (limited report; reports marked as “Qualified” in the CGP FoundationOne CDx examination defined as having the quality‐control status), and cases without any apparent pathogenic mutations (no pathogenic mutations), but identified only with variants of unknown significance (VUS) or clonal hematopoiesis with indeterminate potential (CHIP). Successfully tested cases (STC) were those that were examined without any issues among the 188 cases submitted for CGP testing. Cases with issues such as test failure, limited reports, or no pathogenic mutations were classified as non‐STC.

### Data and statistical analysis

The relationship between each of the preanalytical factors and the CGP test results, including tumor purity assessment, the number of pathogenic mutations, including short variants (base substitution, insertion, and deletion mutations) and copy‐number alterations (CNAs), and the availability of assessments for MSI and TMB, were analyzed by comparing STCs with limited reports and STCs with non‐STCs.

The pathogenicity of each mutation was determined by the institutional molecular tumor board.

We utilized Spearman's rank correlation coefficient (ρ) to analyze the association between each finding and examination results. In the statistical analysis, the *ρ*‐values were calculated by setting STC to 0 and limited report and non‐STC to 1. Statistical analysis was performed using SPSS version 26 (SPSS Inc.), and results with *p* < 0.05 were considered statistically significant.

## RESULTS

In this study, we enrolled 213 participants (113 men and 100 women) with a median age of 64 years. CGP was performed on 152 specimens using FoundationOne® CDx, whereas FoundationOne® CDx Liquid was used for 36 patient specimens. Twenty‐five patient specimens were determined to be non‐submittable for testing based on a preliminary check by the pathologist. The proportion of cases with insufficient tumor cell counts for CGP testing was 45% (19/42) for P‐biopsy, 10% (6/58) for L‐biopsy, 0% (0/67) for P‐ope, and 0% (0/10) for Others. Table 1 presents the distribution of cases by sample type, with 42 cases of P‐biopsy, 58 cases of L‐biopsy, 67 cases of P‐ope, 36 cases of Liquid, and 10 cases categorized as Others. Of the 188 cases successfully subjected to CGP, 32 (17.0%) had limited reports, and 11 (5.9%) revealed no pathogenic mutations. Ductal adenocarcinoma was the most prevalent histological subtype (197 cases), followed by neuroendocrine carcinoma (five cases), acinar cell carcinoma (two cases), adenosquamous carcinoma (two cases), and anaplastic carcinoma (two cases; Table 1). Pathogenic mutations were identified in *KRAS* (155 cases, 82.4%), *TP53* (141 cases, 75.0%), *CDKN2A* (96 cases, 51.1%), *CDKN2B* (57 cases, 30.3%), and *MTAP* (43 cases, 22.9%) (Table 2).

**Table 1:** Clinicopathological findings of the 213 pancreatic cancer cases for whom CGP testing was attempted.

Male/female, *n*	113/100	Postsubmission results	
Age, years^a^	64 (55–71)	STC/Non‐STC, *n*	137/51
FoundationOne/FoundationOne Liquid/NCC	177/36	Pre‐test chemotherapy presence/absence, *n*	117/96
Pancreas biopsy, *n*	42	Therapeutic response	
Liver biopsy, *n*	58	Response (radiology) NE/PD/SD/PR, *n*	17/27/53/15
Pancreas operation, *n*	67	Response (Pathology)Ⅰ/Ⅱa/Ⅱb/Ⅲ, *n* b	10/17/5/1
Liquid, *n*	36	Tumor cell rate (%)^a^	30 (20–40)
Others, *n*	10	Purity assessment^a^	30 (19.4–47.7)
Peritoneum, *n*	5	Number of mutations detected^a^	11 (9–15)
Ovary, *n*	2	Number of pathogenic mutations^a^	4 (3–6)
Omentum, *n*	1	Short variants^c^	3 (2–4)
Small intestine, *n*	1	Copy‐number alterations	0 (0–3)
Lung, *n*	1		

Abbreviations: CGP, comprehensive genomic profiling; STC, cases in which the examination was successful without problems.

^a^ Values are presented as median (interquartile range).

^b^ Evans classification.

^c^ Short variants: base substitution, insertion, and deletion mutations.

**Table 2:** Major pathogenic mutations of CGP testing.

Major pathogenic mutations, *n*			
*KRAS*	155	*GATA6*	13
*KRAS G12D*	73	*KDM6A*	10
*KRAS G12V*	53	*MEN1*	2
*KRAS G12R*	16	*MLL2*	5
*KRAS G12C*	1	*MSH2*	2
*KRAS Q61H*	5	*MSH6*	2
*AKT2*	4	*MTAP*	43
*ARID1A*	11	*MYC*	10
*ASXL1*	2	*NF1*	3
*ATM*	4	*PIC3CA*	4
*BCOR*	1	*RBM10*	5
*BRAF*	4	*RNF43*	13
*BRCA1/2*	1/7	*SMAD4*	37
*CDKN2A/CDKN2B*	96/57	*SMARCA4*	3
*CHEK2*	5	*STK11*	4
*CREBBP*	4	*TET2*	6
*CTNNB1*	3	*TGFBR2*	5
*DNMT3A*	19	*TP53*	141
*FH*	1		

Abbreviation: CGP, comprehensive genomi c profiling.

STC report cases exhibited significantly higher levels of purity assessment (*p* < 0.001, *ρ*: −0.503), MSI availability (*p* < 0.001, *ρ*: 0.758), TMB availability (*p* < 0.001, *ρ*: 0.733), and number of pathogenic mutations (*p* < 0.001, *ρ*: −0.399) than limited report cases. Furthermore, the number of CNAs was notably lower in the limited report cases (*p* < 0.001, *ρ*: −0.367), suggesting that low‐quality samples are unsuitable for the accurate detection of CNAs (Table 3). The factors associated with STC and non‐STC ratios were radiological chemotherapy efficacy assessment (*p* = 0.002, *ρ*: 0.285), aging of samples (*p* < 0.001, *ρ*: 0.274), with a significant difference observed when 500 days were used as the cutoff value (*p* < 0.001, *ρ*: 0.282), and the tumor cell ratio (*p* < 0.001, *ρ*: −0.282; Table 3). No association was observed between formalin fixation time or type of formalin and STC.

**Table 3:** Comparison of STC versus limited, and STC versus non‐STC.

	STC, *n* = 114	Limited, *n* = 30	*ρ*	*p*
Purity assessment*	39.5 (28–55)	20 (10–20)	−0.503	<0.001
MSI, verification success (%)	96	27	−0.758	<0.001
TMB, verification success (%)	98	33	−0.733	<0.001
Number of mutations*	12 (10–15)	10 (8–12)	−0.246	0.003
Number of pathogenic variants*	5.5 (4–7)	3 (2–4)	−0.399	<0.001
Short variants*	3 (2–4)	2.5 (2–3)	−0.177	0.038
Copy‐number alterations*	2 (0–3)	0	−0.367	<0.001

	*n*	STC	non‐STC		
Chemotherapy response (radiology)**	78/33	38/40	6/27	0.285	0.002
Tumor cell ratio (%)	114/38	30 (20–40)	20 (20–30)	−0.282	<0.001
Aging (day)	114/38	205 (43–448)	469 (240–849)	0.274	<0.001
With <499 days/>500 days	114/38	90/24	21/17	0.282	<0.001

*Note*: *Values are presented as median (interquartile range), **NE and PD/SD and PR.

Abbreviations: CGP, comprehensive genomic profiling; MSI, microsatellite instability; STC, cases in which the examination was successful without problems; TMB, tumor mutation burden.

Among the different specimen types, P‐biopsy had the highest STC ratio at 87% (20/23). Additionally, P‐biopsy and L‐biopsy had higher STC ratios than P‐ope. We observed no significant difference in the STC ratio between P‐biopsy and L‐biopsy specimens (*p* = 0.648, *ρ*: 0.054). The lowest rate of neoadjuvant chemotherapy before collection (10%) was found for P‐biopsy, which is commonly used for the definitive diagnosis of pancreatic cancer. The percentage of patients receiving neoadjuvant chemotherapy was 61% for P‐ope and 100% for Liquid and Others (Table 4). The median age was shortest for L‐biopsy at 50 days. P‐biopsy had the highest average tumor cell ratio at 40%, whereas P‐ope had the lowest at 25%. The P‐ope group had the largest tumor area, with a median of 110 mm^2^. The purity assessment of P‐biopsy and L‐biopsy was significantly higher than that of P‐ope (*p* < 0.001). We observed no significant difference in purity assessment between P‐biopsy and L‐biopsy (*p* = 0.848). CNAs could be detected in P‐biopsy, L‐biopsy, and others specimens with a median of 2; however, the median was 0 in P‐ope and Liquid specimens.

**Table 4:** Comparison of P‐biopsy, L‐biopsy, P‐ope, liquid, and other specimen conditions and the results of CGP testing.

*n*	P‐biopsy		L‐biopsy		P‐ope		Others		Liquid	
	42		58		67		10		36	
Pre‐test chemotherapy, *n* (%)	4 (10%)		32 (55%)		41 (61%)		10 (100%)		36 (100%)	
Aging (day)^a^	257 (135–391)	(3, 1106)	50 (10–233)	(3, 826)	528 (395–911)	(115, 1995)	102 (43–367)	(5, 1077)		
Tumor tissue size (mm^2^)^a^	7.85 (4.3–12.0)^b^	(0.2, 60.0)	7.1 (4.7–9.3)	(0.3, 25.0)	110 (55–184.5)	(4.2, 630.0)	73 (40–162)	(9.6, 400.0)		
Tumor cell ratio (%)^a^	40 (30–50)	(10, 90)	30 (29–50)	(1, 90)	25 (20–30)	(15, 65)	30 (20–38)	(20, 55)		
Tumor size (mm^2^) × tumor cell ratio (%)^a^	207 (63–444)	(0, 2100)	310 (125–474)	(0, 2250)	3000 (1440–4753)	(83, 15840)	2040 (1036–4984)	(288, 16000)		
Number of thin slice^a^	33 (29–50)	(15, 80)	30 (22.5–40)	(5, 60)	10 (10–10)	(10, 60)	10 (10–14)	(10, 30)		
Macrodissection, *n* (%)	0 (0%)		8 (14%)		55 (82%)		6 (60%)			
Non‐STC, breakdown										
Not submitted, *n* (%)	19 (45%)		6 (10%)		0 (0%)		0 (0%)		0 (0%)	
Examination impossible, *n* (%)	1 (2%)		4 (7%)		3 (4%)		0 (0%)		0 (0%)	
Limited reports, *n* (%)	2 (5%)		5 (9%)		21 (31%)		2 (20%)		2 (6%)	
No pathogenic mutation, *n* (%)	0 (0%)		0 (0%)		0 (0%)		0 (0%)		11 (31%)	
STC ratio	87%		83%		64%		80%		64%	
Purity assessment^a^	49 (29–55)	(10.0, 97.6)	48 (31–68)	(9.8, 94.0)	22 (17–34)	(9.3, 84.6)	22 (20–45)	(20, 72)	19 (14–27)	(4, 58)
Number of mutations detected, *n* a	11.5 (9.25–15)	(4,60)	12 (11–15)	(6,27)	11 (9–14)	(4,24)	13 (10–15)	(8,22)	10 (7–16)	(3, 27)
Number of pathogenic mutations, *n* a	6 (4–7)	(1, 19)	5.5 (4–7)	(2, 10)	4 (3–6)	(1, 13)	5 (4–6)	(2, 9)	3 (1–4)	(0, 11)
Short variants, *n* a	3 (2–3.75)	(0, 7)	3 (2.5–4)	(0, 9)	3 (2–3)	(1, 9)	3 (2–5)	(2, 5)	3 (1–4)	(0, 8)
Copy‐number alterations, *n* a	2 (0.25–3)	(0, 19)	2 (0–4)	(0, 7)	0 (0–3)	(0, 8)	2 (0–3)	(0, 4)	0 (0–0)	(0, 7)

Abbreviation: CGP, comprehensive genomic profiling; STC, cases in which the examination was successful without problems.

^a^ Values are presented as median (interquartile range: min, max).

^b^ Only P‐biopsy evaluates tissue area.

For P‐biopsy specimens, the median number of cells collected was 3425. The median for STC was 4987, but STC with lower cell counts (2724 [tumor cell ratio: 20%], 2182 [tumor cell ratio: 40%], 1862 [tumor cell ratio: 50%], 1840 [tumor cell ratio: 20%], and 1044 [tumor cell ratio: 30%]) also yielded positive results. The median number of tumor cells deemed unsuitable by the pathologist was 2192 (Table 5). The single case deemed untestable after submission had a cell count of 1317 and a tumor cell ratio of 50%. Cases with limited reports were those with 998 (tumor cell ratio: 15%) and 11417 (tumor cell ratio: 40%) tumor cells. The former case may have yielded a limited report owing to the low number of tumor cells, while inflammatory cell infiltration and necrosis in the latter case, with 11,417 tumor cells, may have been the cause of the limited report (Figure 1g,h). In P‐biopsy, only three cases were unavailable for testing after submission or yielded a limited report.

**Table 5:** Analysis of association between P‐biopsy, L‐biopsy, P‐ope, liquid conditions, and STC ratio, with Spearman correlation coefficients (*ρ*) and *p*‐values for significant correlations (*p* < 0.05).

P‐biopsy, *n* = 42				
Number of tumor cells collected				
Total, *n* = 42^a^	3425 (1630–5021)	(219, 25525)		
STC, *n* = 20^a^	4987 (3844–11097)	(1044, 25525)		
Not submitted, *n* = 19^a^	2192 (1158–3085)	(219, 4788)		
Test impossible, *n* = 1	1317	1317		
Limited reports, *n* = 2^a^	6208 (3603–8812)	(998, 11417)		

	STC	non‐STC	*ρ*	*p*
L‐biopsy, *n* = 58	*n* = 43	*n* = 9		
Aging (day)^a^	17 (9–187)	166 (19–244)	0.214	0.129
Tumor rate (%)^a^	40 (30–60)	20 (10–40)	−0.460	<0.001
Tissue size (mm^2^)^a^	7.5 (5.6–10.9)	6.0 (5.3–7.2)	−0.225	0.112
Tissue (mm^2^) × tumor rate (%)^a^	300 (203–503)	120 (70–265)	−0.372	0.007
Cutoff value is 150 (*n* > 150, %)	36 (84%)	3 (33%)	−0.471	<0.001
P‐ope, *n* = 67	*n* = 43	*n* = 24		
Pre‐test chemotherapy	25 (58%)	16 (67%)	−0.084	0.500
Chemotherapy response (radiology)^b^	2/23	2/14	−0.074	0.646
Chemotherapy response (pathology)^c^	17/2	10/4	0.231	0.195
Aging (day)	503 (287–808)	612 (458–1045)	0.137	0.183
Tumor rate (%)	30 (20–30)	20 (20–30)	−0.155	0.211
Tumor size (mm^2^)	136 (70–206)	90 (25–109)	−0.238	0.052
Tumor size (mm^2^) × tumor rate (%)	3630 (1890–5740)	2200 (875–3105)	−0.257	0.036
Cutoff value is 3000 (*n* > 3000, %)	26 (60%)	7 (29%)	−0.300	0.014
Liquid, *n* = 36	*n* = 23	*n* = 13		
Chemotherapy response (radiology)^b^	18/5	4/9	0.444	0.007
Number of metastasis	2 (1–2)	1 (1–1)	−0.227	0.183
Postchemotherapy period	19 (7–22)	16 (11–24)	−0.071	0.786

Abbreviations: NE, not evaluable; PD, progression; PR, partial response; SD, stable disease; STC, cases in which the examination was successful without problems.

^a^ Values are presented as median (interquartile range).

^b^ NE and PD/SD and PR.

^c^ I and IIa/IIb and III (Evans classification).

For L‐biopsy specimens, a significantly high tendency for STC was observed when the tumor cell ratio was high (*p* = 0.01). Additionally, the STC ratio was significantly higher for cases with a tumor size × tumor cell ratio value > 150 than for those with values < 150 (*p* < 0.001, *ρ*: −0.471; Table 5). Among the five cases with limited reports, three had a tumor cell ratio of <20%. Limited reports were observed in cases with prominent active fibroblast proliferation in the stroma (Figure 1i). Hence, careful examination of the post‐thinning specimen is recommended if the intended number of thin sections cannot be achieved, or if the tumor area significantly decreases after thinning. Similarly, limited documentation was available when the thinning of a specimen resulted in a reduction in the tumor area from 1.6 to 0.2 mm² (Figure 1j).

For the P‐ope specimens, no association between aging, formalin fixation time, type of formalin, or chemotherapy efficacy determination and STC ratio was observed. Although tumor size and tumor cell ratio alone were not associated with the STC ratio, the STC ratio was significantly higher for cases with a tumor size × tumor cell ratio value >3000 than for those with values <3000 (*p* = 0.014, *ρ*: −0.300; Table 5). Postchemotherapy cases showed cellular degeneration, fibrosis, and histiocytic infiltration, which may have been caused by treatment. However, no statistically significant difference in the tumor‐cell ratio was observed between the groups that received preoperative chemotherapy and those that did not.

Liquid specimens did not reveal any association between the number of metastatic sites and the STC ratio. Although we included 10 NE cases and the number of analyses was small, the results of this study indicate that cases with NE or PD chemotherapy responses determined using radiographic imaging had a higher STC ratio than those with SD or PR (*p* = 0.007; Table 5).

## DISCUSSION

Our findings suggest that cellularity and the tumor‐cell ratio are critical factors influencing the success of CGP testing.

Pancreatic cancer often invades and grows by intertwining with existing pancreatic tissues, incorporating various cell types, such as background pancreatic acinar cells, smooth muscle cells, and glandular epithelial cells of the duodenal mucosa. Assessing the absolute tumor cell ratio in such cases becomes challenging and less objective. We did not have sufficient cases to allow statistical analysis, but we included cases whose specimens contained many cells, other than tumor cells, and thus obtained limited reports for the CGP test. Although it is difficult to objectively assess tumor cell ratios in such cases, we may empirically alert the attending physicians that a limited report for CGP examination is likely when the tumor cell ratio of the available specimen is approximately 20%, but lymphocyte, histiocyte, or fibroblast proliferation is prominent in the background.

P‐biopsy tends to have a lower mixture of background tissue and higher ratio of tumor cells than P‐ope, rendering it a more favorable specimen for CGP testing. However, the number of tumor cells collected is often low, with a median of 3425 cells. We found that limited reports may occur even with a cell count of 11 417, likely owing to high inflammatory cell infiltration within the necrosis. There was STC even given a cell count as low as approximately 2000 (with a minimum of 1044 cells). Typically, the required DNA for an NGS panel test is ≥10 ng, while the amount obtained from one nucleated diploid cell is estimated at 6 pg, which corresponds to approximately 2000 cells.^13^ As preoperative chemotherapy for pancreatic cancer has become common, P‐biopsy and cytology specimens can be used to collect tumor cells before chemotherapy‐induced degeneration, which is crucial for genetic testing. The importance of P‐biopsy specimens at the time of diagnosis is expected to increase, as gene retrieval using cytological specimens has become feasible in recent years.^14^, ^15^, ^16^

L‐biopsy specimens had a higher number of tumor cells than P‐biopsy specimens and showed fewer stromal cells and other cells intercalated with tumor cells than those in P‐ope specimens, causing a trend toward a significantly higher tumor cell ratio (*p* < 0.001, *ρ*: −0.337). The shorter aging duration observed in L‐biopsy than in other specimens is likely attributable to its biopsy for CGP test submission. Contrary to P‐biopsy and P‐ope, which use existing specimens collected for diagnostic or therapeutic purposes, obtaining biopsy samples for CGP testing holds inherent risks. Liver biopsy also presents risks that vary based on the collection site and condition of the patient; the attending physician must therefore be consulted before performing a re‐biopsy for CGP testing.^17^ L‐biopsies are performed in approximately half of the cases before chemotherapy and in cases with high chemotherapy efficacy and a high degree of tumor cell degeneration and fibrosis, which may contribute to limited reports. While specimens categorized as Others were not numerous enough for statistical analysis, their STC ratio was comparable with that of P‐biopsy and L‐biopsy.

Liquid specimens possess the advantage of being able to be submitted when no other specimens are available or when re‐biopsy involves a high risk or is impractical. Notably, in the liquid specimens, no cases were deemed ineligible for testing after submission. However, in 31% of the cases, the only genetic mutations identified were VUS or suspected CHIP in the expert panel review, and no pathogenic mutations were detected. The numbers of identified pathogenic mutations (*p* < 0.001, *ρ*: −0.380) and CNAs (*p* < 0.001, *ρ*: −0.389) were significantly lower than those in the P‐biopsy and L‐biopsy specimens.

Unlike biopsy specimens, surgical specimens are subject to several conditions, including longer ischemic time and time required for formalin to penetrate the center of the specimen, which render them unsuitable for CGP testing. We observed no significant differences between the STC ratio and factors, such as formalin fixation time and type of formalin fixation.

The STC ratio demonstrated a significant change with aging, with 500 days as the cutoff value. However, some cases remained STC‐viable beyond 1000 days, highlighting the need to assess the storage conditions of tissue blocks in the future. Among the cases in this study, we identified a case fixed in 10% unbuffered formalin for 144 h and aged 1995 days at the time, which was successfully tested. Conversely, some cases yielded limited reports even when all pre‐analytic conditions were optimal. With the exception of aging, a wide variety of pre‐analysis factors may be associated with gene quality; however, we could not identify any association between pre‐analysis conditions and the CGP test STC ratio in this study. Factors, such as cold ischemic time, formalin fixation time, type of formalin, and aging, reportedly significantly impact gene quality.^8^, ^9^, ^18^

**Figure 2:**
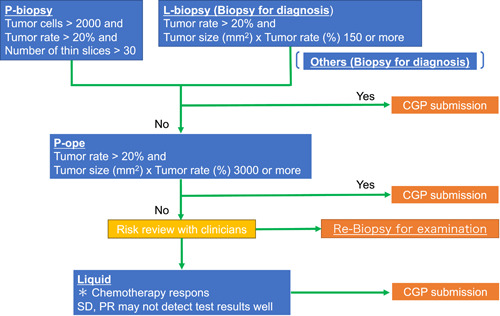
Proposed follow‐up chart for CGP test submission in pancreatic cancer cases. CPG, comprehensive genomic profiling.

Based on our findings, we propose a draft flowchart (Figure 2) for submitting pancreatic cancer specimens for CGP tests. P‐biopsy is the first candidate in cases where the tumor cell count is >2000, the tumor cell ratio is >20%, and a number of achievable thin slices is ≥30. However, in cases where the tumor area is small, increasing the number of thin slices to be submitted is appropriate; our results suggest an average of 37 slices. Liver biopsy is also considered the first candidate in cases with liver metastases, a tumor cell ratio >20%, and a tumor size (mm²) × tumor cell rate (%)>150, as it offers better conditions than P‐biopsy. In cases where other metastatic site specimens fulfill better conditions than P‐/L‐biopsy specimens, they can be considered for submission. In cases where these specimens do not meet the requirements, P‐ope material should be considered. In cases where the tumor cell ratio is >20% and the tumor size × tumor cell ratio is >3000, they should be considered candidates for submission. In the event that these specimens also do not meet the requirements, consultation with a pathologist and clinician is advised to discuss whether to submit a liquid biopsy or perform a re‐biopsy. These suggestions based on our experience may need to be adapted if the sensitivity and specificity of the CGP test change in the future.

## CONCLUSIONS

The implementation of CGP testing for pancreatic cancer may be subject to limitations attributable to the challenges of obtaining an adequate number of tumor cells, particularly with surgical material, which can be further impacted by prior chemotherapy. Despite a lower quantity of cells collected, P‐biopsy demonstrated a higher success rate in producing specimens suitable for CGP than P‐ope and is therefore recommended as the preferred method to generate specimens. However, further research is needed on an enhanced number of cases, as numerous preanalytic conditions are intricately involved in the success of CGP testing. As the demand for biomarker testing rises, pathologists must possess a comprehensive understanding of specimen characteristics and work closely with clinicians and laboratory technicians to make appropriate specimen selections.

## AUTHOR CONTRIBUTIONS

Kota Washimi (first author and corresponding author) reviewed the data and literature, analyzed the results, performed the histological diagnosis, and wrote the manuscript. Yukihiko Hiroshima provided resources and expertise in genetic interpretation and treatment strategies. Makoto Ueno, Satoshi Kobayashi, and Naoto Yamamoto analyzed clinical findings. Yohei Miyagi provided resources and expertise for genetic analysis and revised the manuscript. Shinya Sato, Chie Hasegawa, Emi Yoshioka, Kyoko Ono, and Yoichiro Okubo reviewed the histology. Tomoyuki Yokose provided the first and corresponding authors with pancreatic carcinoma findings and differential diagnosis and revised the manuscript.

## CONFLICTS OF INTEREST STATEMENT

Tomoyuki Yokose and Yohei Miaygi are Editorial Board members of Pathology International and co‐authors of this article. To minimize bias, they were excluded from all editorial decision‐making related to the acceptance of this article for publication. None declared for the other authors. The authors declare no conflicts of interest.

## ETHICS STATEMENT

This study was approved by the Research Ethics Review Committee of Kanagawa Cancer Center (approval no. 2022 epidemiology‐133). This investigation was conducted in accordance with the Declaration of Helsinki. We used the opt‐out method to obtain patient consent for this study.

## Data Availability

The data sets used and/or analyzed during the current study are available from the corresponding author upon reasonable request.
